# Comparison of prevalence, clinical evolution and vaccination rate against COVID 19 in a population of patients diagnosed with Dual Bipolar disorder and Non-dual bipolar disorder

**DOI:** 10.1192/j.eurpsy.2022.1037

**Published:** 2022-09-01

**Authors:** B. Losilla Rodríguez, F. Palermo Zeballos, M. Ortiz Jiménez

**Affiliations:** Hospital Universitario Virgen Macarena, Psychiatry, Sevilla, Spain

**Keywords:** covid 19, dual disorder, Prevalence, bipolar disorder

## Abstract

**Introduction:**

Since the beginning of the pandemic, 4,745,519 cases, 396,878 hospitalizations and 82,884 deaths with COVID-19 have been reported in Spain. As of August 24, 2021, 76.4% of Andalusians over 12 years of age have the complete vaccination regimen.

**Objectives:**

Main: to calculate the prevalence of COVID 19 infection, clinical evolution, and vaccination rate in a population of patients diagnosed with dual bipolar disorder. Secondary: compare these data with those obtained inpatients diagnosed with non-dual bipolar disorder.

**Methods:**

Retrospective descriptive study. The study population is made up of patients diagnosed with dual bipolar disorder and non-dual bipolar disorder (according to DSM 5 criteria). Infection, admission, death, and vaccination data were obtained from the patient’s medical history.

**Results:**

Of the 7 patients diagnosed with dual bipolar disorder, the prevalence of COVID 19 infection, since the beginning of the pandemic is 0% and of the 21 patients diagnosed with non-dual bipolar disorder the prevalence is 9.51% (2/21). Of the patients with COVID 19 infection, none required hospital admission and no deaths occurred. The vaccination rate in the group of patients with dual bipolar disorder is 85,71% (6/7) and in the group of non-dual bipolar disorder is 61.91% (13/21), no finding statistically significant differences between both groups.

**Conclusions:**

In our study the prevalence of COVID 19 infection inpatients diagnosed with dual bipolar disorder is 0% and the vaccination rate is 85.71%. While in patients with non-dual bipolar disorder the prevalence is 9.51%, there were no admissions, no deaths and the vaccination rate is 61.91%.

**Disclosure:**

No significant relationships.

